# Dynamics of the HCl + C_2_H_5_ Multichannel Reaction on a Full-Dimensional Ab Initio Potential Energy Surface

**DOI:** 10.1021/acs.jpca.4c02042

**Published:** 2024-05-29

**Authors:** Kitti Horváth, Viktor Tajti, Dóra Papp, Gábor Czakó

**Affiliations:** MTA-SZTE Lendület Computational Reaction Dynamics Research Group, Interdisciplinary Excellence Centre and Department of Physical Chemistry and Materials Science, Institute of Chemistry, University of Szeged, Rerrich Béla tér 1, Szeged H-6720, Hungary

## Abstract

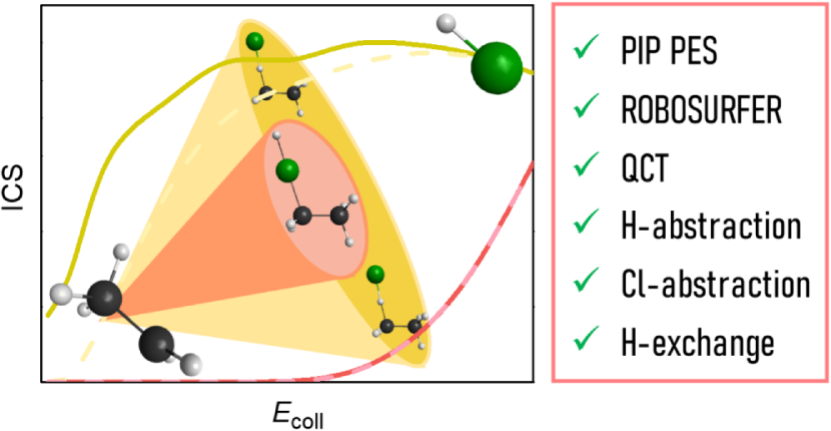

We report a full-dimensional ab initio analytical potential energy surface (PES), which accurately describes the HCl + C_2_H_5_ multichannel reaction. The new PES is developed by iteratively adding selected configurations along HCl + C_2_H_5_ quasi-classical trajectories (QCTs), thereby improving our previous Cl(^2^P_3/2_) + C_2_H_6_ PES using the Robosurfer program package. QCT simulations for the H’Cl + C_2_H_5_ reaction reveal hydrogen-abstraction, chlorine-abstraction, and hydrogen-exchange channels leading to Cl + C_2_H_5_H’, H’ + C_2_H_5_Cl, and HCl + C_2_H_4_H’, respectively. Hydrogen abstraction dominates in the collision energy (*E*_coll_) range of 1–80 kcal/mol and proceeds with indirect isotropic scattering at low *E*_coll_ and forward-scattered direct stripping at high *E*_coll_. Chlorine abstraction opens around 40 kcal/mol collision energy and becomes competitive with hydrogen abstraction at *E*_coll_ = 80 kcal/mol. A restricted opening of the cone of acceptance in the Cl-abstraction reaction is found to result in the preference for a backward-scattering direct-rebound mechanism at all energies studied. Initial attack-angle distributions show mainly side-on collision preference of C_2_H_5_ for both abstraction reactions, and in the case of the HCl reactant, H/Cl-side preference for the H/Cl abstraction. For hydrogen abstraction, the collision energy transfer into the product translational and internal energy is almost equally significant, whereas in the case of chlorine abstraction, most of the available energy goes into the internal degrees of freedom. Hydrogen exchange is a minor channel with nearly constant reactivity in the *E*_coll_ range of 10–80 kcal/mol.

## 1 Introduction

The theoretical dynamical investigation of systems containing more than six atoms on full-dimensional analytical potential energy surfaces (PESs) has only become feasible in the recent decade(s).^1−33^ Following the extensive literature on atom + H_2_O/CH_4_ reactions,^34−47^ the atom + C_2_H_6_ reactions have emerged as the new benchmark systems,^8−14^ which represent several new possibilities and also many challenges for reaction dynamics studies: (1) rules of thumb discovered for small/medium-sized reactions can be extended for polyatomic reactivity, (2) these reactions often involve multiple competitive reaction channels, calling for sophisticated PES development techniques, and (3) with the increasing number of degrees of freedom, mode- and bond-selectivity are of major interest in these complex systems. Many of such investigations have enriched our knowledge in recent years, using quasi-classical trajectory (QCT) computations. Following initial QCT studies of atom + ethane reactions on force-field-based PESs,^10,11^ high-quality ab initio potential energy surfaces have been developed by our group for different reactions of ethane, using an automated improvement strategy,^48^ providing excellent agreement with dynamics experiments regarding the HF/HCl product rotational and/or vibrational distributions for the F and Cl + C_2_H_6_ reactions,^8,9^ and mode-specific vibrational populations of H_2_O in the OH + ethane reaction.^49^ Other polyatomic reactions, such as O(^3^P) + C_2_H_4_,^2^ OH + CH_4_,^3^ H/F/Cl/OH + CH_3_OH,^4−7^ OH^–^ + CH_3_F/CH_3_I,^16,21,32^ F^–^(H_2_O)/Cl^–^(H_2_O) + CH_3_I,^17^ Cl + propene/pentane,^18,19^ F^–^/OH^–^ + CH_3_CH_2_Cl,^20,24^ NH_2_^–^ + CH_3_I,^22^ HBr/HI + C_2_H_5_,^25,26^ H_2_O/NH_3_ + CH_2_OO,^27,33^ F/Cl + CH_3_NH_2_,^28,29^ and F^–^ + (CH_3_)_3_CI,^31^ have also been the subjects of QCT simulations, and vibrational and rotational mode-specificity have been explored in several of these postsix-atomic systems. Additionally, reduced-dimensional quantum dynamics computations have also been performed for such polyatomic reactions.^50−52^

Specifically, following the studies of the HBr + CH_3_^53−55^ and F/Cl(^2^P_3/2_) + C_2_H_6_^8−11^ systems, the HX + C_2_H_5_ [X = Br and I] reactions have been investigated in detail in our group.^25,26,56−58^ The kinetics of these processes have long been subjects of interest due to their submerged barriers and the resulting non-Arrhenius behavior.^59−66^ Yin and Czakó have developed high-quality ab initio spin–orbit-corrected PESs^25,26^ for these reactions using the ManyHF procedure^67^ and the Robosurfer program,^48^ and studied their dynamics to gain an in-depth picture behind the unusual kinetics. Both reactions have two main product channels: H-abstraction (HA), leading to X(^2^P_3/2_) + C_2_H_6_, and X-abstraction (XA), producing H + C_2_H_5_X.^56,57^ HA is exothermic both for X = Br and I and proceeds through a shallow prereaction minimum and a submerged transition state close in energy, while the endothermic XA route has a positive energy barrier and a shallow postreaction well.^56,57,68^ QCT simulations show larger HA reactivity and an increased forward-scattering preference of the products when X = I, as well as H-side and side-on preference for HX and C_2_H_5_, respectively, for both reactions.^25,26,56,57^ The X-abstraction and hydrogen-exchange (only for X = Br) channels, as well as vibrational mode-specificity, have also been investigated in these reactions.^56,57^ These PESs have then been further improved to describe the competition of the HA and XA channels at collision energies up to 80 kcal/mol, as well.^58^ In contrast to H-abstraction, XA prefers the backward-scattering direct rebound mechanism with a more likely collision-energy transfer into the internal degrees of freedom of the products.^58^ HBr-excitation promotes all three reaction pathways, while exciting the HI vibration affects mainly the I-abstraction route.^58^ HX vibrational enhancement turned out to be much more efficient than translational excitation, whereas vibrational excitation of C_2_H_5_ does not have a significant effect on reactivity; sometimes it even causes inhibition.^58^

Here, we aim to study the dynamics of the HCl + C_2_H_5_ reaction by improving our previous Cl(^2^P_3/2_) + C_2_H_6_ PES^8^ to describe the relevant channels of the backward reaction, including also higher-energy regions up to 80 kcal/mol. We investigate the dependence of reactivity on the attack angle of the reactants, along with the product scattering-angle distributions, and follow the energy transfer through the different mechanisms of the title reaction.

## 2 Computational Details

We further improve the full(21)-dimensional ab initio potential energy surface previously developed in our group in 2020 for the Cl(^2^P_3/2_) + C_2_H_6_ reaction^8^ using the Robosurfer program package^48^ to be able to investigate the backward reaction HCl + C_2_H_5_. We also extend the energy range of the PES to higher energies to follow four possible channels of the title reaction: (1) hydrogen-abstraction leading to C_2_H_6_ + Cl(^2^P_3/2_); (2) chlorine-abstraction resulting in C_2_H_5_Cl + H; (3) methyl-substitution (CH_3_Cl + CH_3_); and (4) hydrogen-exchange (H’Cl + C_2_H_5_ → C_2_H_4_H’+ HCl).

In this work, we identify two novel stationary points of the PES: a transition state (TS) and a prereaction minimum for Cl-abstraction, using the same levels of theory as for the previously found^68^ H-abstraction stationary points: the final geometries are optimized at the explicitly-correlated ROHF-UCCSD(T)-F12b/aug-cc-pVTZ^69,70^ level of theory, and to obtain the benchmark relative energies of these two stationary points, we perform single-point energy computations at the ROHF-UCCSD(T)-F12b/aug-cc-pVQZ level and further include post-(T) (at the UHF-UCCSDT/cc-pVDZ^71^ and UHF-UCCSDT(Q)/cc-pVDZ^72^ levels) as well as core-correlation (ROHF-UCCSD(T)/aug-cc-pwCVTZ)^73^ energy contributions. For all the electronic-structure computations, the Molpro program package^74^ is used, except for the post-(T) contributions, which are determined with the MRCC program.^75,76^

For computing the new energy points, we apply the same composite electronic structure level of theory as used for the initial PES,^8^ which is based on the UCCSD(T)-F12b method^69^ with an augmented double-ζ basis set improved by a triple-ζ basis-set correction obtained with the explicitly-correlated second-order Mo̷ller–Plesset perturbation method,^77^ and a spin–orbit term determined with the multireference configuration interaction (MRCI) method^78^ with a minimal active space of 5 electrons on 3 spatial 3p-like orbitals using the Breit–Pauli operator in the interacting-states approach,^79^ and also including Davidson-correction (+Q),^80^ which estimates higher-order correlation contributions. The final composite energy is obtained as
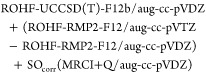
1

The fitting of the points is carried out using the monomial symmetrization approach (MSA) of the permutationally invariant polynomial method.^81^ For the fitting, we apply the Morse-like variables *y*_*ij*_ = exp(−*r*_*ij*_/*a*), where *r*_*ij*_ are the interatomic distances and the *a* parameter, set to 1.5 bohr, controls the asymptotic behavior of the PES, and we use the *E*_0_/(*E* + *E*_0_) weighing factor, where *E* is the actual energy relative to the global minimum of the fitting set and *E*_0_ = 0.04 hartree. The energy points are fitted using a least-squares procedure, and the fifth-order function applied requires 3234 fitting coefficients.

The development with Robosurfer^48^ starts from the initial PES,^8^ on which we run QCT simulations and gain new geometries from the problematic points of the trajectories. These structures are then subjected to various similarity checks before they are judged to be worthy of electronic structure computations, which are carried out automatically using Molpro.^74^ The points with the largest fitting errors are added to the fitting set, as these are the most likely to improve the quality of the PES. This process is repeated until the desired accuracy of the PES is reached.

During the Robosurfer iterations, the quasi-classical trajectories on the actual PESs are initiated from the HCl + C_2_H_5_ direction at 1, 5, 10, 20, 30, 40, 50, 60, 70, and 80 kcal/mol collision energies (*E*_coll_). The orientation of the reactants is random and their distance is , where *x* = 29 bohr and the impact parameter *b* is varied in the interval 0.00–5.75 bohr with a step size of 0.25 bohr. 96 (4 at each *b*) trajectories are run in every Robosurfer iteration. The final PES consists of 26 064 energy points, which means 14363 new geometries with respect to the 2020 PES.

The final QCT simulations are run at the same collision energies as those used for the PES development with *x* = 16.0 bohr and *b* ∈ [0.0, *b*_max_], where *b*_max_ is the impact parameter value where reaction probability vanishes and the step size of *b* is 0.5 bohr. We propagate 1000 trajectories with a time step of 0.0726 fs for each *b*−*E*_coll_ pair. A trajectory stops when the largest final atom–atom distance becomes 1 bohr larger than the largest initial one.

Integral cross-section (ICS) values (σ) for the different reaction channels are computed by a numerical integration of the *P*(*b*) opacity functions (the *P* reaction probability as a function of *b*):
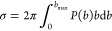
2

Zero-point energy(ZPE)-restricted ICS values are also obtained for the H-abstraction and the Cl-abstraction cannels, where those trajectories are discarded in which the final classical vibrational energy of the molecular product is below its harmonic ZPE. The scattering angle distributions of the products are calculated by binning the cosine of the included angle θ of the relative velocity vectors of the center of masses (COMs) of the reactants and the products into 5 equidistant bins. Cos(θ) = −1 (θ = 180°) corresponds to backward scattering. Initial attack-angle distributions are computed for both the C_2_H_5_ and the HCl fragments: in the first case, the attack angle is defined as the included angle of the vector pointing from the C atom of the CH_3_ group to the C atom of the CH_2_ group and the velocity vector of the COM of the C_2_H_5_ moiety, while in the case of the HCl reactant, the attack angle is the included angle of the vector pointing from Cl to H and the velocity vector of the COM of the HCl. Then, the cosines of these angles are also binned into five equidistant bins.

## 3 Results and Discussion

**Figure 1:**
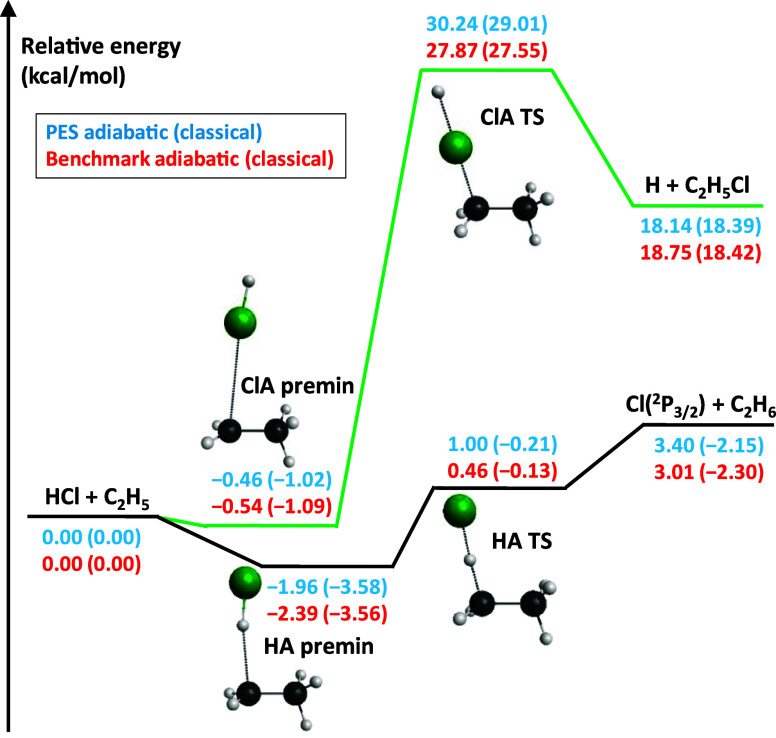
Schematic potential energy surface of the HCl + C_2_H_5_ reaction showing the adiabatic and classical relative energies of the stationary points along the hydrogen-abstraction (HA) and chlorine-abstraction (ClA) pathways. The PES values correspond to the present analytical surface, and the benchmark data are obtained at the CCSD(T)-F12b/aug-cc-pVQZ + δ[CCSDT] + δ[CCSDT(Q)] + Δcore + ΔSO + ΔZPE level of theory, where ΔZPE is applied for the adiabatic energies.

We have further improved the potential energy surface recently developed in our group for the Cl(^2^P_3/2_) + C_2_H_6_ reaction^8^ using the Robosurfer program package,^48^ which was used for the original PES development as well. We have applied the same composite electronic structure method (see eq 1) to compute the energies of the selected new geometries, and we have added a total of 14 363 points, which results in a PES containing 26 064 energy points altogether. As shown in Table 1, the root-mean-square (rms) error of the improved PES is below 1 kcal/mol within the first 40 kcal/mol range above the global minimum (which is at −3.58 kcal/mol energy relative to the reactants), which covers the relevant stationary points of the title reaction (see Figure 1). The rms error falls even below 0.5 kcal/mol in the 0–20 kcal/mol interval relative to the global minimum, and in the 40–80 kcal/mol range, it still does not exceed 2 kcal/mol.

**Table 1:** Root-Mean-Square Errors and the Number of Energy Points in Different Relative-Energy Intervals with Respect to the Global Minimum (HA Premin, −3.58 kcal/mol Relative to the Reactants) of the Newly Developed PES

energy range (kcal/mol)	0–20	20–40	40–60	60–80	80–100	>100
RMS error (kcal/mol)	0.41	0.86	1.33	1.88	2.73	5.71
number of points	1628	8360	6972	6399	2552	153

In Figure 1, we show the schematic potential energy diagram of the H-abstraction (HA) and Cl-abstraction (ClA) pathways of the title reaction. The stationary points of the HA pathway were already identified in a previous comprehensive study on the energetics of the X + C_2_H_6_ [X = F, Cl, Br, I] reactions.^68^ The benchmark structures and energies of the premin and transition state involved in the ClA channel are newly identified in this work, at the same level of theory as used for the HA stationary points^68^ (see Computational Details). As can be seen in Figure 1, the Cl-abstraction route is endothermic with a 19 kcal/mol 0 K reaction enthalpy and has a relatively high energy barrier at about 28 kcal/mol relative to the reactants. The HA channel, in contrast, is barrierless (−0.13 kcal/mol) when the ZPE contribution is not taken into account but has a very small adiabatic barrier (0.46 kcal/mol). The HA pathway is adiabatically also endothermic with a 0 K reaction enthalpy of 3.01 kcal/mol, but is slightly exothermic (−2.30 kcal/mol) if we exclude ZPE. Both channels feature prereaction minima: that of ClA is only slightly below the reactant asymptote; however, the HA premin has a –3.6 kcal/mol classical depth. For the HA channel, several postreaction minima could also be identified at about 1.0–1.8 kcal/mol below the HA product asymptote.^68^ The stationary points of the methyl-substitution (MS) pathway are not shown in Figure 1 due to their higher relative energies and smaller relevance in the dynamics. The MS reaction can occur via two mechanisms: through either a Walden-inversion or front-side attack transition state, at classical (adiabatic) energies of 31.6(34.7) or 54.4(57.6) kcal/mol relative to the reactants of the title reaction.^68^

**Figure 2:**
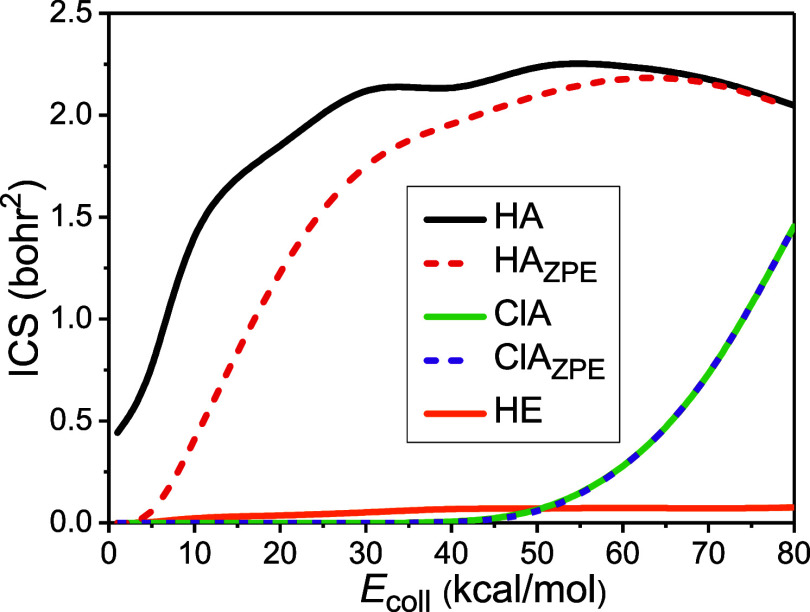
Cross-sections as a function of collision energy for the hydrogen-abstraction (HA), chlorine-abstraction (ClA), and hydrogen-exchange (HE) channels of the HCl + C_2_H_5_ reaction. ZPE subscripts denote zero-point energy constraints.

In Figure 2, we present the integral cross sections (ICSs) as a function of collision energy (i.e., excitation functions) for the H- and Cl-abstraction, as well as for the H-exchange channel of the title reaction. The HA channel shows reactivity at all collision energies applied, in accordance with its 1 kcal/mol adiabatic barrier, while the ClA pathway opens only above 40 kcal/mol, well above its 30.24 kcal/mol adiabatic barrier height on the PES, due probably to steric reasons (see below). The H-exchange process is also present in the whole energy range studied, although with very low “reactivity”. The ClA reaction becomes competitive with HA at the highest collision-energy range. Interestingly, the excitation functions of the HA and ClA reactions have distinctly different shapes: the ClA reaction shows a so-called concave-up^82−84^ shape, while the HA function is of a concave-down silhouette with a maximum at 50 kcal/mol (it starts to decrease as the ClA channel opens). The shape of the excitation function is proposed^82^ to reflect how tight the bending potential is at the saddle point, that is, in how far steric effects are significant in the reaction: the concave-up increase of the ICS with increasing collision energy can be linked to a tight-bend transition state, where the collision requires a special orientation of the reactants, which was e.g., seen for the O(^3^P) + CH_4_ reaction,^46^ while the concave-down form, characteristic e.g., in the case of the Cl + CH_4_^42−46^ and F + C_2_H_6_^9^ reactions, is related to a loose-bend transition state, implying orientation-independent barrier. The methyl-substitution pathway, leading to CH_3_Cl + CH_3_, is not observed in the collision-energy range studied, probably due to the high steric specificity required in the process and/or due to its supposedly multistep nature.

We also apply a zero-point-energy constraint for the ICSs to treat the well-known artificial ZPE leakage in QCT calculations: the molecular products should have higher classical vibrational energy than their harmonic ZPE, otherwise, the trajectory is discarded. As seen in Figure 2, ZPE-restriction affects significantly the reactivity of the H-abstraction reaction, especially at low collision energies; however, leaves the shape of the excitation functions unchanged. In exothermic reactions, the reaction energy usually compensates and thus masks the artificial flow of vibrational energy into the translational or into other vibrational modes; however, in the case of this slightly endothermic channel, the loss of vibrational energy is more pronounced. On the other hand, at higher collision energies, the initial translational energy seems to counteract ZPE-leakage in the HA reaction. At the same time, from Figure 2, it is clear that the ZPE constraint does not have any effect on the reactivity of the much more endothermic Cl-abstraction channel. This can be caused by a series of reasons: (1) in this case, the ZPE of the two reactant species, which is very unlikely to drastically leak into the translational degree of freedom, cumulates in the forming C_2_H_5_Cl fragment; (2) the relative translational energy of the products is small, because their reduced mass is very close to that of the forming H atom; and (3) this translational energy is mostly “taken” by the light H atom, which has much larger velocity than C_2_H_5_Cl. This way, enough internal energy remains in the C_2_H_5_Cl product to avoid ZPE-violation.

**Figure 3:**
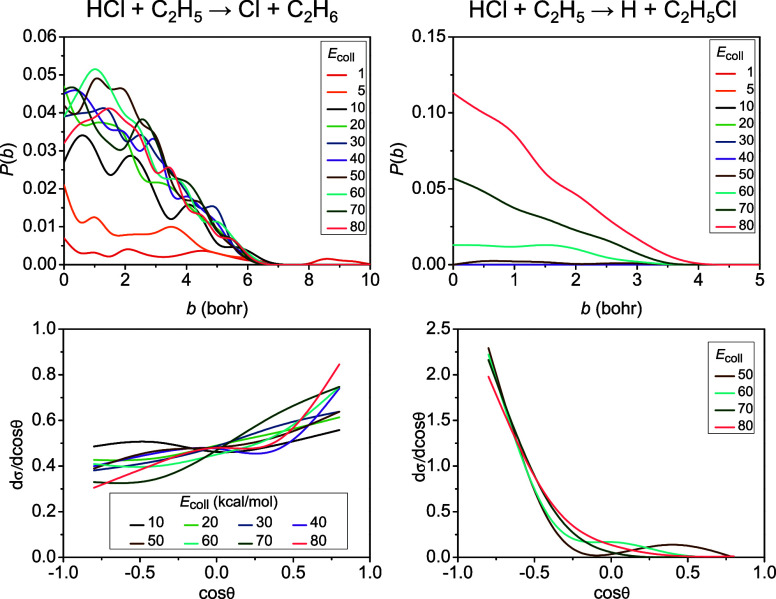
Reaction probabilities as a function of impact parameter and scattering angle distributions for the hydrogen-abstraction (left) and chlorine-abstraction (right) channels of the HCl + C_2_H_5_ reaction at different collision energies given in kcal/mol.

In Figure 3, we show opacity functions (reaction probabilities as a function of the impact parameter) along with product scattering angle distributions for both of the main channels. The probability of the H-abstraction reaction is increasing with increasing collision energy, from 0.5% to 4.5% at the zero impact parameter. The HA reaction features the largest maximum value of the impact parameter (*b*_max_ = 10 bohr) at the lowest collision energy, where the collision time is long enough to allow the reactants to build interaction at a larger distance, while the *b*_max_ value is significantly lower, 7 bohr at all the other energies. The scattering angle distributions of the HA products indicate isotropic scattering at lower energies (the two lowest energies are excluded due to the low reaction probability) and an increasing preference for forward scattering as collision energy increases. The isotropic pattern suggests a complex-formation-mediated indirect mechanism where the colliding fragments lose information about their incident directions. The slightly favored forward direction at higher energies indicates a shift to a direct stripping mechanism, where a somewhat increased HA probability is seen at larger *b* values, which support the stripping of the H atom. In contrast, in the case of the Cl-abstraction channel, which opens only at 50 kcal/mol collision energy, we see an order of magnitude larger probabilities with respect to those of HA, enhanced with increasing collision energy, from 1–2% to 12% at *b* = 0. This reaction exhibits much smaller *b*_max_ values (3.5–4 bohr), which are in accordance with the tight-bend nature of its transition state, suggested by the concave-up excitation function, as a tight-bend barrier gives rise to a narrower cone of acceptance, and thus a smaller distance range, where the reactants are able to develop interaction.^82−84^ This restricted opening of the cone of acceptance with increasing collision energy prevents large-*b* collisions, thus, leads to backward-scattered products even at higher energies, formed in small-*b* collisions, in excellent agreement with the ClA scattering angle distributions.

**Figure 4:**
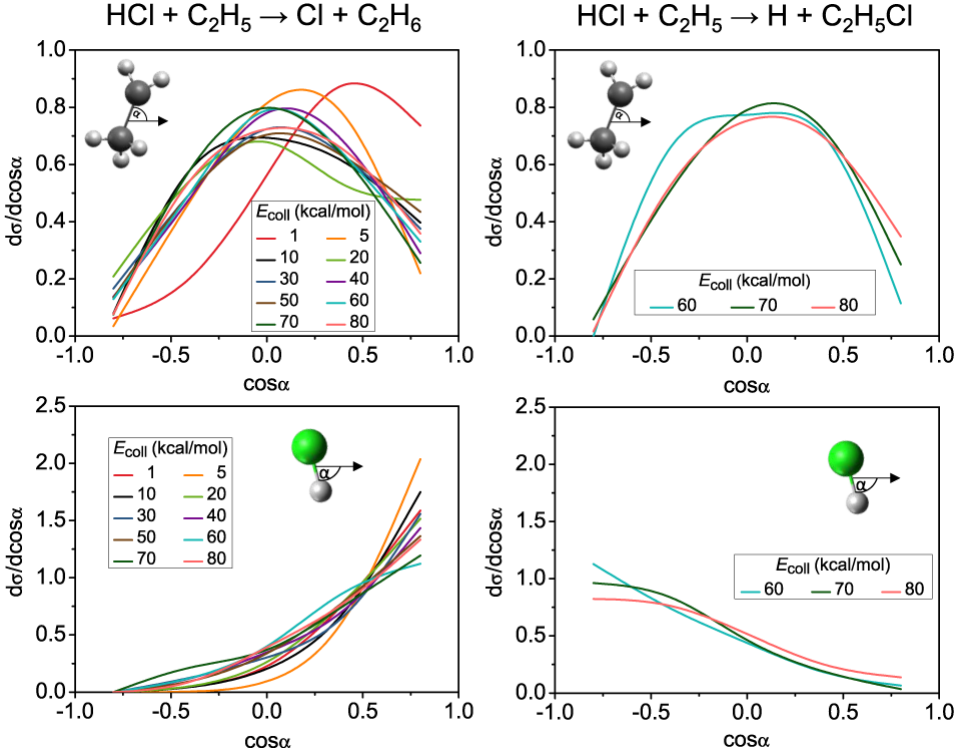
Distributions of the C_2_H_5_ (upper panels) and HCl (lower panels) initial attack angles for the hydrogen-abstraction (left) and chlorine-abstraction (right) channels of the HCl + C_2_H_5_ reaction at different collision energies.

The initial attack-angle distributions are also extracted from QCT calculations and are shown in Figure 4. In case of the ethyl radical the distributions have maxima at 90° attack angle, except the two lowest collision energies, where the maxima are shifted toward smaller angles, showing increased CH_2_–side preference at low energies. In the case of the Cl-abstraction reaction, the ethyl radical also prefers side-on collisions, with a wider angle-range (a plateau in the distribution) at 60 kcal/mol collision energy, and with maxima slightly shifted at higher energies, indicating a minor CH_2_–side preference. The HCl fragment clearly favors H-side collisions in the case of the H-abstraction reaction, and Cl-side approach, when the Cl atom is abstracted by the ethyl radical.

If we compare these results to those obtained for the HX + C_2_H_5_ [X = Br, I] reactions,^25,26,58^ we find for the H-abstraction cannel that as the halogen size increases, both the forward scattering preference of the products and the CH_2_–side collision preference of the ethyl radical reactant increase. In the case of halogen abstraction, we see backward scattered products and similar attack-angle preferences in all three X = Cl, Br, and I reactions.

**Figure 5:**
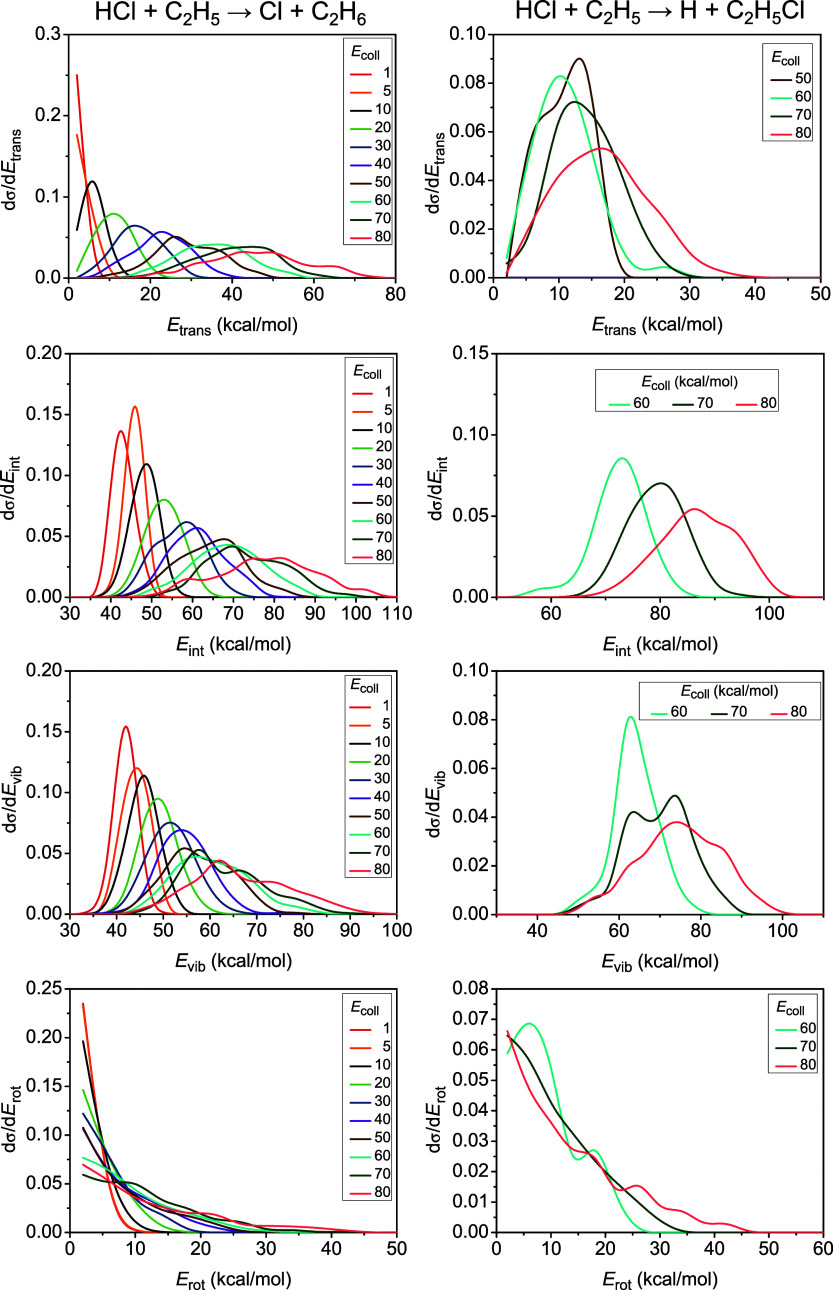
Product relative translational, internal, vibrational, and rotational energy distributions (from up to down) for the hydrogen-abstraction (left) and chlorine-abstraction (right) channels of the HCl + C_2_H_5_ reaction at different collision energies given in kcal/mol.

The postreaction energy flow is also investigated in the title reaction: Figure 5 shows the relative translational-energy distributions of the products for the HA and ClA channels, along with the internal energy distributions of the C_2_H_6_ and the C_2_H_5_Cl products, decomposed into rotational and vibrational energies. The initial available energy (collision energy + reactant ZPEs – reaction energy) flows almost equivalently into the translational motion of the products and into the internal degrees of freedom of ethane during the H-abstraction reaction, as both the maxima of the translational and those of the internal energy distributions shift by around the half of the collision energy increment. However, the vibrational modes of ethane store more energy than its rotational motion. In contrast, in the Cl-abstraction reaction, the initial available energy is mostly fueled into the internal degrees of freedom of ethyl chloride, with a considerable amount of energy exciting vibrations (the maxima of the vibrational distributions blue-shift almost with the increment of the collision energy) and somewhat less energy converting into rotation. It is also clear that no ZPE-violation is observed (the ZPE of C_2_H_5_Cl on the PES is 41.51 kcal/mol) in the ClA reaction, as already seen in Figure 2. Also, our assumption on the ZPE-preservation in the molecular product seems to be reasonable, not only for the ClA but for the HA reaction as well, as a similar amount of vibrational excitation is found in the case of the molecular products of the two reactions at the three highest collision energies. Additionally, the smaller translational energy for the ClA products (H + C_2_H_5_Cl) with respect to those of the HA channel (Cl + C_2_H_6_) caused by the difference in their reduced masses is also clearly seen from Figure 5. Very similar energy-transfer tendencies are observed for all three halogens, Cl, Br,^25^ and I.^26^

As to the hydrogen-exchange process, from visually inspecting the corresponding QCT trajectories, 124 altogether, we can distinguish between two different mechanisms. One mechanism starts with the CH_2_ group abstracting a H atom, after which the (original) CH_3_ group turns toward the Cl atom, which then abstracts a H back from this CH_3_ unit. The other mechanism involves an H-exchange between the CH_2_ unit and the HCl molecule, while the CH_3_ unit does not play a role. The first scenario happens more often; the other one, involving only the CH_2_ group, makes only 12% of the HE trajectories and is favored at higher collision energies.

## 4 Summary and Conclusions

We investigate the dynamics of the HCl + C_2_H_5_ reaction for the first time by running quasi-classical trajectories using the improved version of the full-dimensional ab initio analytical potential energy surface previously developed for the Cl(^2^P_3/2_) + C_2_H_6_ reaction,^8^ which now includes higher energy ranges and describes the “backward” reaction accurately. In the 1–80 kcal/mol collision energy range, we identify three reaction paths: hydrogen abstraction leading to Cl + C_2_H_6_, chlorine abstraction resulting in H + C_2_H_5_Cl, and hydrogen exchange (H’Cl + C_2_H_5_ → HCl + C_2_H_4_H’). The complex methyl substitution (HCl + C_2_H_5_ → CH_3_Cl + CH_3_) process is not observed in the studied collision-energy range. We also find two novel stationary points, a transition state and an entrance-channel minimum, involved in the Cl-abstraction reaction, for which we determine benchmark geometries and energies as well. The QCT simulations show that H-abstraction and H-exchange are present throughout the 1–80 kcal/mol energy span, the first being dominant and the latter being only of minor reactivity. The Cl-abstraction channel opens only at 50 kcal/mol, well above its 30 kcal/mol adiabatic barrier height, due to the special steric demands of the reactive collision, which are also explained in the present study, and becomes more and more competitive with HA as collision energy increases. An interesting difference between the excitation functions of HA and ClA is observed: HA reactivity shows a concave-down shape with increasing collision energy, indicating a loose-bend activation barrier, while the excitation function of ClA has a concave-up rise, reflective of a tight-bend transition state. This tightness prevents the opening of the cone of acceptance of the ClA reaction as the initial energy increases, thus, constraining it to proceed by small-impact-parameter collisions, resulting in the backward scattering of the products. In contrast, the product scattering angle distributions of the HA reaction are mainly isotropic as a signal of an indirect mechanism at low energies, and slightly favor the forward direction, indicative of a direct stripping mechanism, at higher energies. H-exchange turns out to proceed via two different mechanisms: H-abstraction by the CH_2_ unit from HCl followed by H abstraction from the (original) CH_3_ group by Cl, or exchange of H atoms between the CH_2_ group and HCl. From the distributions of the attacking angles, we find a side-on preference in both abstraction processes in the case of the ethyl radical, while a H/Cl-side preference is observed for HCl in the H/Cl-abstraction reactions. During the HA reaction, the available energy splits almost equivalently between the relative translational motion of the products and the internal energy of ethane, stored mostly in the vibration of the molecular product. On the contrary, during Cl-abstraction, only a small part of the initial energy is taken by product recoil, and the majority of the increment in the collision energy excites the internal motions of C_2_H_5_Cl, especially flowing into vibration. That is why the products of the markedly endothermic ClA reaction do not show signs of ZPE-violation, whereas ZPE-restriction has a significant impact on the reactivity of HA, featuring only a slight endothermicity. We hope that our study can be a motivation for new experiments studying the dynamics and the interesting steric behavior of the different pathways of the title reaction.

## Supporting Information Available

Numerical data behind the integral cross-sections and the opacity functions obtained from the QCT calculations (PDF)
