# Supplementary material for: Dynamics of the HCl + C2H5 Multichannel Reaction on a Full-Dimensional Ab Initio Potential Energy Surface
Source: J Phys Chem A. 2024 May 29;128(22):4474–82. doi: 10.1021/acs.jpca.4c02042 (PMC11163425; doi:10.1021/acs.jpca.4c02042)
Supplement: Supplementary file 1 — jp4c02042_si_001.pdf [file jp4c02042_si_001.pdf]

SUPPORTING INFORMATION

Dynamics of the HCl + C_2H_5 Multichannel Reaction on a Full-Dimensional Ab Initio Potential Energy Surface

Kitti Horváth, Viktor Tajti, Dóra Papp,* and Gábor Czakó*

MTA-SZTE Lendület Computational Reaction Dynamics Research Group, Interdisciplinary Excellence Centre and Department of Physical Chemistry and Materials Science, Institute of Chemistry, University of Szeged, Rerrich Béla tér 1, Szeged H-6720, Hungary

*E-mail: dorapapp@chem.u-szeged.hu and gczako@chem.u-szeged.hu

Integral cross sections and reaction probabilities obtained from QCT calculations

Table S1. Integral cross sections ($bohr^2$) as a function of collision energy (kcal/mol) for the different product channels with and without ZPE-restrictions.

E_{coll}	H-exchange(HE)	H-abstraction (HA)	Cl-abstraction (ClA)	HA ZPE	ClA ZPE
1	0	0.44296	0	0	0
5	0	0.67858	0	0	0
10	0.02827	1.58336	0	0.39741	0
20	0.03456	1.82998	0	1.28648	0
30	0.05027	2.19754	0	1.81270	0
40	0.07226	2.08445	0	1.95564	0
50	0.06912	2.26980	0.03770	2.10487	0.02827
60	0.07540	2.24624	0.24033	2.19440	0.24033
70	0.06912	2.19126	0.67387	2.17869	0.67387
80	0.07540	2.04832	1.45456	2.03261	1.45456

Table S2. Reaction probabilities for the H-abstraction channel at different *b* impact parameters (bohr) and collision energies (kcal/mol).

b	1	5	10	20	30	40	50	60	70	80
**0**	0.007	0.021	0.027	0.047	0.039	0.045	0.042	0.039	0.046	0.032
**0.5**	0.002	0.008	0.036	0.035	0.040	0.047	0.037	0.046	0.048	0.038
**1**	0.004	0.015	0.032	0.038	0.040	0.041	0.053	0.054	0.035	0.038
**1.5**	0.001	0.007	0.020	0.037	0.043	0.034	0.044	0.047	0.032	0.043
**2**	0.005	0.008	0.030	0.036	0.029	0.037	0.049	0.038	0.028	0.037
**2.5**	0.003	0.008	0.028	0.021	0.037	0.026	0.035	0.038	0.042	0.036
**3**	0.002	0.008	0.018	0.022	0.028	0.039	0.037	0.022	0.033	0.020
**3.5**	0.002	0.011	0.009	0.021	0.025	0.014	0.020	0.023	0.022	0.030
**4**	0.003	0.008	0.018	0.016	0.019	0.020	0.016	0.022	0.023	0.012
**4.5**	0.004	0.004	0.013	0.012	0.013	0.014	0.018	0.009	0.017	0.015
**5**	0.003	0.003	0.004	0.010	0.018	0.012	0.008	0.013	0.007	0.006
**5.5**	0.002	0.002	0.005	0.005	0.003	0.004	0.005	0.007	0.008	0.008
**6**	0.001	0.002	0.004	0.002	0.002	0.003	0.001	0.003	0.001	0.002
**6.5**	0.001	0	0.001	0	0	0	0	0	0	0
**7**	0	0	0	0	0	0	0	0	0	0
**7.5**	0	0	0	0	0	0	0	0	0	0
**8**	0	0	0	0	0	0	0	0	0	0
**8.5**	0.002	0	0	0	0	0	0	0	0	0
**9**	0.001	0	0	0	0	0	0	0	0	0
**9.5**	0.001	0	0	0	0	0	0	0	0	0
**10**	0	0	0	0	0	0	0	0	0	0

Table S3. Reaction probabilities for the Cl-abstraction channel at different *b* impact parameters (bohr) and collision energies (kcal/mol).

b	50	60	70	80
**0**	0	0.013	0.057	0.113
**0.5**	0.003	0.013	0.049	0.099
**1**	0.002	0.011	0.036	0.090
**1.5**	0.002	0.014	0.031	0.056
**2**	0	0.011	0.022	0.048
**2.5**	0.001	0.004	0.017	0.030
**3**	0.001	0.002	0.007	0.017
**3.5**	0	0	0	0.005
**4**	0	0	0	0
